# Vitamin K3 and vitamin C alone or in combination induced apoptosis in leukemia cells by a similar oxidative stress signalling mechanism

**DOI:** 10.1186/1475-2867-11-19

**Published:** 2011-06-10

**Authors:** Angelica R Bonilla-Porras, Marlene Jimenez-Del-Rio, Carlos Velez-Pardo

**Affiliations:** 1 School of Medicine, Medical Research Institute, University of Antioquia (UdeA), Calle 62 # 52-59, Building 1, Laboratory 411/412; SIU- Medellin, Colombia

## Abstract

**Background:**

Secondary therapy-related acute lymphoblastic leukemia might emerge following chemotherapy and/or radiotherapy for primary malignancies. Therefore, other alternatives should be pursued to treat leukemia.

**Results:**

It is shown that vitamin K3- or vitamin C- induced apoptosis in leukemia cells by oxidative stress mechanism involving superoxide anion radical and hydrogen peroxide generation, activation of NF-κB, p53, c-Jun, protease caspase-3 activation and mitochondria depolarization leading to nuclei fragmentation. Cell death was more prominent when Jurkat and K562 cells are exposed to VC and VK3 in a ratio 1000:1 (10 mM: 10 μM) or 100:1 (300 μM: 3 μM), respectively.

**Conclusion:**

We provide for the first time *in vitro *evidence supporting a causative role for oxidative stress in VK3- and VC-induced apoptosis in Jurkat and K562 cells in a domino-like mechanism. Altogether these data suggest that VK3 and VC should be useful in the treatment of leukemia.

## Background

Leukemia is a cancer of the bone marrow and blood. The cause of this hematological disorder is currently still unknown. Today, first-line therapy for the treatment of leukemia includes chemotherapy and radiotherapy. Unfortunately, secondary therapy-related acute lymphoblastic leukemia might emerge following chemotherapy and/or radiotherapy for primary malignancies [1]. Therefore, other therapeutic alternatives based on the reactivation of the apoptotic program should be pursued to eliminate cancer cells [2]. Accordingly, the use of vitamin K3 (VK3, also known as menadione [3]) and vitamin C (VC, also known as sodium ascorbate [4]) alone or in combination (VK3: VC [5]) is highly promising in cancer treatment. Yet, the precise pathway(s) by which VK3 and/or VC induce leukemia cell death are not well established. Moreover, given the complexity of death pathways within a cell, placing these pathways in the proper relationship to the drug trigger is challenging.

During the last three decades, vitamin K3 has been known to display anti-tumor action both *in vivo *and *in vitro *in human cancer cell lines [4]. Several observations suggest that vitamin K3 might induce apoptosis -a type of cell death- [6] through different biochemical routes including severe depletion of glutathione and sulfhydryl-containing proteins and alteration of intracellular Ca^2+ ^homeostasis [7], activation of c-Jun NH_2_-terminal kinase (JNK, [8]), activation of Fas/Fas ligand system independently of the pro-apoptotic p53 protein [9], activation of Fas-dependent and Fas-independent pathways [10] and NF-κB activation [11]. Because most studies have looked at a given pathway in isolation using different cell types, the potential interaction between pathways have often not been addressed. Therefore, the complete mechanism(s) of cell death signalization induced by vitamin K3 in a single cell model remains unclear.

Vitamin C is a water-soluble vitamin effective as antioxidant compound under normal conditions [12]. However, Chen and collaborators [13,14] have shown that pharmacological concentrations of (5-15 mM) vitamin C was pro-oxidant, generating H_2_O_2_-dependent cytotoxicity toward a variety of cancer cells *in vitro *and *in vivo *without adversely affecting normal cells. Interestingly, it has been shown that (10 μM) VK3 in combination of (2 mM) VC kill leukemia cells (e.g., K562 cells) by oxidative stress independent of caspase-3, with a minor percentage of cell displaying mitochondrial depolarisation and DNA fragmentation without chromatin condensation consistent with a necrosis-like cell death [15]. Yet, the mechanism by which vitamin C-induced apoptosis in Jurkat and K562 cells is not yet fully established.

Since it is known that the transcription factor NF-κB [16], p53 [17], c-Jun [18] and caspase-3 [19] are involved in apoptosis signaling, we hypothesized that VK3 and VC might induce cell death in leukemia cells through activation of such factors by oxidative stress. To test this assumption, we sought to investigate the molecular mechanism by which VC, VK3 alone or in combination (ratio 100:1 [20]) induce cell death in Jurkat (clone E06-1) and K562 leukemia cell lines and lymphocyte cells in relation to the aforementioned pro-apoptotic transcription factors and caspase-3. Although, the role of vitamin C as therapeutic compound is still controversial [21], understanding the mechanism of vitamins alone or together may provide insight into more effective anti-cancer therapy.

## Results

### Vitamin K3 (VK3) and vitamin C (VC) alone or in combination induce apoptosis in Jurkat and K562 cells through anion superoxide radical (O_2_^.-^)/H_2_O_2 _generation and mitochondrial damage

**Figure 1:**
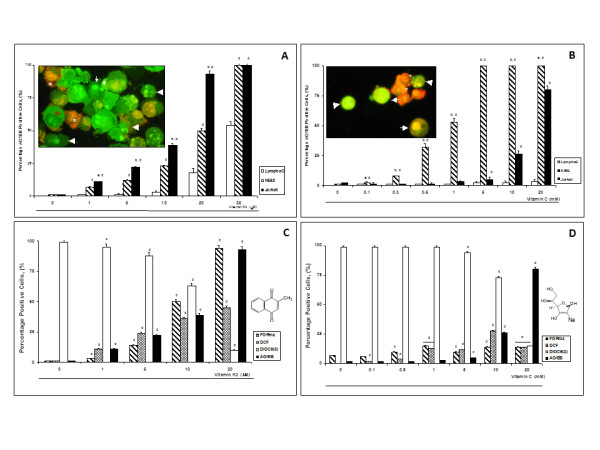
**Vitamin K3 (VK3) and vitamin C (VC) produce reactive oxygen species, mitochondrial depolarization and nuclear morphology indicative of apoptosis in lymphocyte and leukemia cells**. Lymphocyte, Jurkat and K562 were incubated with increasing concentration of VK3 (A) and VC (B) for 24 h. Nuclear morphological changes was evaluated using AO/EB staining. *Inset*: Representative fluorescent photomicrography shows cell shrinkage and rounding, chromatin condensation (*arrows*), nuclei fragmentation (*arrowheads*) indicative of apoptosis and necrotic cells (*asterisk) *in K562 cells treated with (35 μM) VK3 (A) and (1 mM) VC (B). The ANOVA showed significantly differences among the three cells groups, *p *< 0.0001; *post-hoc *comparison showed significantly increase in cell death morphology in the leukemia cells on each VK3 and VC concentration versus lymphocytes. Jurkat cells were incubated with (10 μM) VK3 (C) and (10 mM) VC (D) for 24 h. Cells were evaluated for O_2_^.-^/H_2_O_2 _production, Δψ_m _and nuclear morphological changes indicative of apoptosis. NBT^+ ^stained blue-purple precipitate cells, DCF^+ ^green fluorescent cells, DiOC_6_(3)^high/low+ ^green fluorescent cells and apoptotic nuclei percentage is expressed as mean of percentage (%) ± S.D. from three independent experiments. ANOVA test for each condition showed differences among groups *p *< 0.0001. The *post-hoc *comparison showed increase in number of DCF, AO/EB and NBT^+ ^cells, whereas the number of DiOC_6_(3)^+ ^cells decreased in a concentration-dependent fashion. One-way ANOVA analysis with Bonferroni *post-hoc *analysis was performed. A *p*-value of ^a^*p *< 0.05 and ^b^*p *< 0.001 Jurkat versus K562 or ^c^*p *< 0.05 and ^d^*p *< 0.001 versus lymphocytes (A, B) or control (C, D) was considered significant.

Figure 1A and 1B show that VK3 and VC provoked typical morphological features of apoptosis in a concentration-dependent fashion according to the conventional AO/EB staining technique [22]. However, (early/late) apoptosis and necrosis (~20%-50%) morphology of cell death were observed at high VK3 (e.g., 35 μM for both cells) and VC (e.g., 20 mM for Jurkat and > 1 mM for K562) concentrations (*insets*). Remarkably, K562 cells were the most sensible to VC, whereas both cell lines were more sensible to VK3 than lymphocytes (e.g. Jurkat and K562 cells displayed ~40% & ~25% AO/EB positive nuclei whereas lymphocytes displayed about 3% AO/EB positive nuclei when exposed to 10 μM VK3). These results prompted us to fully examine the effect of VK3 and VC on Jurkat cell line.

**Figure 2:**
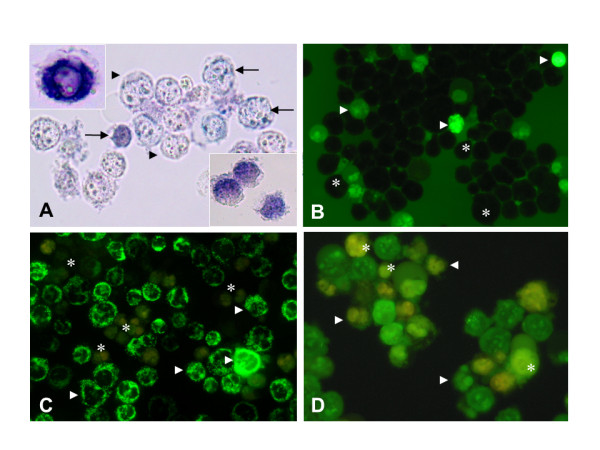
**Vitamin K3 (VK3) generates anion superoxide radicals (O_2_^.-^), hydrogen peroxide (H_2_O_2_), mitochondrial depolarization and apoptotic morphology in Jurkat cells. Jurkat cells (1 × 10^6 ^cells/mL) were incubated with (10 μM) VK3 at different interval as indicated below**. (A) Representative light photomicrography showing NBT^+ ^stained blue-purple precipitate cells (i.e. formazan, *arrows*) and NBT^- ^stained cells (i.e. translucent cells, *arrowheads*) indicative of positive and negative (O_2_^.-^) production, respectively from Jurkat cells incubated with VK3 for 3 h at 37 °C. *Insets*: Magnification of four NBT^+ ^cells showing cytoplasmic blue-purple precipitate from Jurkat cells incubated with VK3 for 24 h at 37 °C. (B) Representative fluorescent photomicrography (ex. 450-490 nm, em. 515 nm) illustrating DCF^+ ^(green bright light, *arrowheads*) and DCF^- ^(dark round-shape, *asterisks*) fluorescent stained cells indicative of positive and negative H_2_O_2 _production, respectively. (C) Representative fluorescent photomicrography (ex. 450-490 nm, em. 515 nm) illustrating DiOC_6_(3)^+ ^green fluorescent (*arrowheads*) stained cells indicative of high-polarized and low-polarized mitochondria and DiOC_6_(3)^- ^(non-fluorescent, *asterisks*) stained cells indicative of depolarized mitochondria. (D) Representative fluorescent photomicrography (ex. 450-490 nm, em. 515 nm) showing typical nuclear apoptotic morphology such as highly condensed chromatin (*asterisks*) and nuclear fragmentation (*arrowheads*) from Jurkat cells treated with (10 μM) VK3. Magnification A: 1000x (*inset *1200x); B: 600x; C: 800x; D: 1000x.

Figure 1C shows that VK3 produced both anion superoxide radical (O_2_^.-^) and H_2_O_2 _in a concentration-dependent fashion up-to 20 μM, e.g. 94% and 43% percent of formazan (Figure 2A) and DCF positive (Figure 2B), respectively in Jurkat cells. Figure 1C also shows that increasing concentrations of VK3 induced mitochondrial depolarization, as reflected by DiOC_6_(3) non-fluorescent cells (Figure 2C) in a concentration-dependent fashion. Interestingly, apoptotic morphological alterations (Figure 2D) were associated with mitochondrial depolarization. Since high VK3 concentrations (e.g. > 35 μM) induced nuclear and cytoplasmic destruction of cells (necrosis), thus avoiding a clear-cut evaluation of apoptotic morphology (data not shown), 10 μM VK3 was selected for further experiments.

Recently, it has been shown that mM concentrations of ascorbate were cytotoxic to neuroblastoma cells mediated by H_2_O_2 _[23]. Figure 1D shows that VC was capable to generate either O_2_^.- ^or H_2_O_2 _at the indicated concentrations. Moreover, VC was moderately or highly toxic to Jurkat and K562 cells, respectively at high concentrations. Of note, whilst 1 mM VC induced apoptotic/necrotic morphology in K562, 10 mM concentration induces apoptosis in Jurkat to a similar extend provoked by (10 μM) VK3. Interestingly, VC was innocuous for lymphocyte cells at the indicated concentrations.

### A ratio 1000: 1 and 100: 1 VC: VK3 provoke apoptosis in Jurkat cells and K562 cells, respectively

Table 1 shows that a ratio 100:1 (VC: VK3) did not affect Jurkat cells, but this ratio (300 VC: 3VK3) induced (~30%) apoptosis in K562, according to AO/EB and DiOC_6_(3) staining techniques. A ratio 1000:1 resulted in an effective toxic effect in Jurkat cell line.

**Table 1:** Effect of different ratio combination of Vitamin K3 (VK3) and vitamin C (VC) in Jurkat cells.

Treatment/Assay	AO/EB (%)	DiOC_6_(3)^High/Low ^(%)
Untreated	< 1 ± 0	> 99 ± 0
VK3 (1 μM)	1 ± 1	98 ± 1
VC (100 μM)	1 ± 0	99 ± 1
VK3 (1 μM) + VC (100 μM)	1 ± 1	98 ± 1
VK3 (2 μM)	1 ± 1	98 ± 1
VC (200 μM)	1 ± 1	98 ± 2
VK3 (2 μM) + VC (200 μM)	2 ± 1	97 ± 1
VK3 (3 μM)	5 ± 1^a^	96 ± 1
VC (300 μM)	4 ± 1^a^	97 ± 1
VK3 (3 μM) + VC (300 μM)	2 ± 1^a^	97 ± 2
VK3 (4 μM)	11 ± 1^a^	87 ± 3^a^
VC (400 μM)	< 1 ± 0^c^	> 99 ± 0^c^
VK3 (4 μM) + VC (400 μM)	8 ± 1^a,b^	92 ± 2^a,b,c^
VK3 (5 μM)	22 ± 1^a^	73 ± 2^a^
VC (500 μM)	2 ± 1^c^	97 ± 1^c^
VK3 (5 μM) + VC (500 μM)	10 ± 2^a,b,c^	88 ± 2^a,c^
VC (5000 μM)	3 ± 1	97 ± 2^b,c^
VK3 (5 μM) + VC (5000 μM)	48 ± 2^a,b,c^	68 ± 2^a,b,c^
VK3 (10 μM)	35 ± 3^a^	60 ± 2^a^
VC (10000 μM)	26 ± 2^a^	75 ± 3^a,c^
VK3 (10 μM) + VC (10000 μM)	58 ± 3^a,b,c^	63 ± 3^a,b^

Jurkat cells (1 × 10^6 ^cell/mL) were left untreated or treated with VC or VK3 alone or in combination at the indicated concentration for 24 h. After this time, mitochondrial membrane potential and nuclear morphological changes were evaluated using DiOC_6_(3) and acridine orange/ethidium bromide (AO/EB) staining, as described in *Materials and Methods*. High polarized and low-polarized mitochondria (green fluorescent DiOC_6_(3) high/low positive cells) and positive apoptotic nuclei percentage is expressed as mean of percentage (%) ± S.D. from three independent experiments. A one-way ANOVA analysis was performed, *p *< 0.0001. ^a^Significantly different from untreated by Bonferroni *post-hoc *analysis for DiOC_6_(3) and Games-Howell *post hoc *comparison for AO/EB. ^b^Significantly different from VC by Bonferroni *post-hoc *analysis for DiOC_6_(3) and Games-Howell *post hoc *comparison for AO/EB. ^c^Significantly different from VK3 by Bonferroni *post-hoc *analysis for DiOC_6_(3) and Games-Howell *post hoc *comparison for AO/EB.

### VK3 and VC alone or in combination induce apoptosis through of reactive oxygen species

CP55,940 [24] and N-acetyl-cysteine (NAC) antioxidants dramatically reduced apoptosis to almost control values and importantly abrogated H_2_O_2 _when cells where treated with VK3, VC alone or VK3 in combination with VC (Table 2). A similar protective effect was observed when K562 cells were exposed to (20 μM) VK3, (0.5 mM) VC and VC: VK3 in a ratio 300:3 and antioxidants (data not shown).

**Table 2:** Hydrogen peroxide (H_2_O_2_) is involved in vitamin K3 (VK3) and vitamin C (VC) toxic effect on Jurkat leukemia cells.

Treatment/Assay	AO/EB (%)	DCF (%)
Untreated	< 1 ± 0	2 ± 1
VC (10 mM)	26 ± 2	28 ± 2
VK3 (10 μM)	35 ± 3	39 ± 3

VK3 (10 μM) + VC (10 mM)	48 ± 3	55 ± 3
CP55,940 (10 nM)	< 1 ± 0	1 ± 1
CP55,940 (10 nM) + VC (10 mM)	1 ± 1^b^	1 ± 1^b^
CP55,940 (10 nM) + VK3 (10 μM)	2 ± 1^a^	8 ± 2^a^
CP55,940 (10 nM) + VC (10 mM) + VK3 (10 μM)	3 ± 1^c^	14 ± 2^c^
NAC (1 mM)	< 1 ± 0	1 ± 0
NAC (1 mM) + VC (10 mM)	2 ± 1^b^	7 ± 2^b^
NAC (1 mM) + VK3 (10 μM)	4 ± 1^a^	12 ± 2^a^
NAC (1 mM) + VC (10 mM) + VK3 (10 μM)	7 ± 1^c^	20 ± 2^c^

Jurkat cells (1 × 10^6 ^cell/mL) were left untreated or treated with VC (10 mM) or VK3 (10 μM) alone or with VC (10 mM): VK3 (10 μM) in the absence or presence of the antioxidant CP55,940 (10 nM) and N-acetyl-cysteine (NAC, 1 mM) at 37 °C for 24 h. After this time, nuclear morphological changes and hydrogen peroxide (H_2_O_2_) generation were evaluated using acridine orange/ethidium bromide (AO/EB) and 2',7'-dichlorofluorescein diacetate (DCFH_2_-DA) staining, as described in *Material and Methods*. Positive apoptotic nuclei and green fluorescent dichlorofluorescein (DCF) positive cells percentage is expressed as mean of percentage (%) ± S.D. from three independent experiments. A one-way ANOVA analysis was performed, *p *< 0.0001. ^a^Significantly different from VK3 by Bonferroni *post-hoc *analysis for DCF and Games-Howell *post hoc *comparison for AO/EB. ^b^Significantly different from VC by Bonferroni *post-hoc *analysis for DCF and Games-Howell *post hoc *comparison for AO/EB. ^c^Significantly different from VK3-VC by Bonferroni *post-hoc *analysis for DCF and Games-Howell *post hoc *comparison for AO/EB.

### VK3 and VC alone or in combination induce apoptosis in Jurkat and K562 cells associated with NF-κB, p53 and c-Jun transcription factors and caspase-3 activation

**Figure 3:**
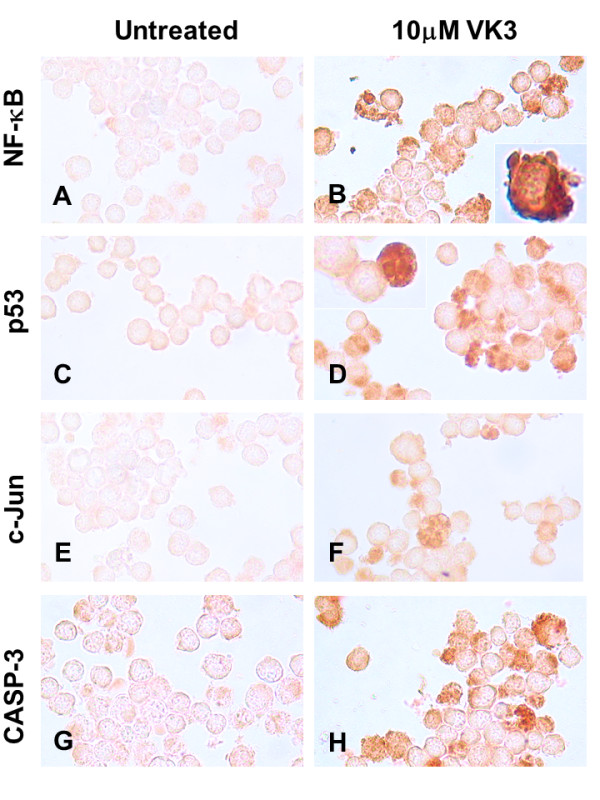
**Vitamin K3 (VK3) alone induces simultaneous activation of the transcription factor NF-κB, p53, c-Jun and protease caspase-3 in Jurkat cells**. Jurkat cells were left untreated (A, C, E, G) or exposed to (10 μM) VK3 (B, D, F, H), at 37 °C for 24 h. After this time of incubation, cells were stained with anti-NF-κB-p65 (A, B), anti-p53 (C, D), anti-c-Jun (E, F) and anti-caspase-3 (G, H) antibodies according to procedure described in *Materials and Methods*. Notice that NF-κB, p53, c-Jun and caspase-3 (CASP-3) positive-nuclei (dark brown color) reflect their nuclear translocation/activation and appear to correlate with the apoptotic nuclear morphology, i.e. condensed/fragmented nuclei compared with untreated cells (A, C, E, G) or cytoplasmic activation (brown color). Magnification 660x (A-H). *Inset *Magnification (B, 2000x; D:1100x).

**Figure 4:**
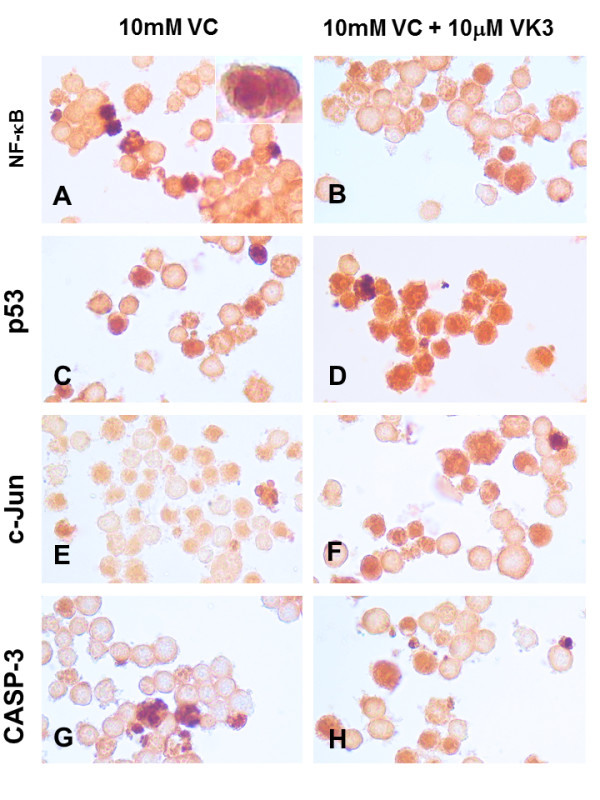
**Vitamin C (VC) alone or in combination with vitamin K3 (VK3: VC) induce simultaneous activation of the transcription factor NF-κB, p53, c-Jun and protease caspase-3 in Jurkat cells**. Jurkat cells were exposed to (10 mM) VC (A, C, E, G) and (10 μM) VK3 plus (10 mM) VC (B, D, F, H) for 24 h. After this time of incubation, cells were stained with anti-NF-κB-p65 (A, B), anti-p53 (C, D), anti-c-Jun (E, F) and anti-caspase-3 (G, H) antibodies according to procedure described in *Materials and Methods*. Notice that NF-κB, p53, c-Jun and caspase-3 (CASP-3) positive-nuclei (dark brown color) reflect their nuclear translocation/activation and appear to correlate with the apoptotic nuclear morphology, i.e. condensed/fragmented nuclei compared with untreated cells (Figure 3 A, C, E, G) or cytoplasmic activation (brown color). Magnification 660x (A-H). *Inset *Magnification (A: 2000x).

Table 3 shows that all the specific pharmacological inhibitors reduced almost completely VK3- and VC-induced apoptosis and mitochondrial damage effect compared to control values, but moderately reduced cell death and Δψ_m _when cells were exposed in combination (~30-44% reduction). To confirm the participation of NF-κB, p53, c-Jun and caspase-3 in VK3, VC and VK3 plus VC-induced apoptosis, we performed immunocytochemical assessment. As illustrated in Figure 3 B, D, F, and H, Jurkat cells incubated with 10 μM VK3 clearly showed DAB^+ ^nuclei staining of the active form of NF-κB (~36%), p53 (~38%), c-Jun (~40%) and CASP-3 (~40%) compared to untreated cells (1-2% DAB^+ ^nuclei), where inactive transcription factors and protease reside in the cytoplasm (Figure 3 A, C, E and G). In addition, immunocytochemical assessment of cells treated with VC alone showed DAB^+ ^nuclei staining of NF-κB (~18%), p53 (~15%), c-Jun (~15%) and CASP-3 (~16%) (Figure 4 A, C, E and G, respectively) compared to untreated cells. Not surprisingly, treated cells with (10 μM) VK3 in combination with (10 mM) VC stained DAB^+ ^for NF-κB (~38%, Figure 4B), p53 (~75%, Figure 4D), c-Jun (~48%, Figure 4F) and caspase-3 (~47%, Figure 4H) compared to untreated cells. Likewise, K562 cells treated with (500 μM) VC, (20 μM) VK3 and (300:3) VC/VK3 showed DAB^+ ^nuclei staining for NF-κB, p53, c-Jun and CASP-3 to a similar extend as Jurkat cells (data not shown).

**Table 3:** NF-κB, p53, c-Jun and caspase-3 molecules are involved in vitamin K3 (VK3)- and vitamin C (VC)-induced apoptosis in Jurkat cells.

Treatment/Assay	AO/EB (%)	DiOC_6_(3)^High/Low^ (%)
Untreated	< 1 ± 0	> 99 ± 0
VK3 (10 μM)	35 ± 3	63 ± 2
VC (10 mM)	26 ± 2	73 ± 3
VK3 (10 μM) + VC (10 mM)	58 ± 3	63 ± 3

PDTC (10 nM)	< 1 ± 0	> 99 ± 0
PDTC (10 nM) + VK3 (10 μM)	3 ± 1^a^	97 ± 1^a^
PDTC (10 nM) + VC (10 mM)	8 ± 2^b^	90 ± 2^b^
PDTC (10 nM) + VK3 (10 μM) + VC (10 mM)	13 ± 2^c^	80 ± 3^c^
PFT (50 nM)	< 1 ± 0	> 99 ± 0
PFT (50 nM) + VK3 (10 μM)	4 ± 1^a^	98 ± 1^a^
PFT (50 nM) + VC (10 mM)	3 ± 1^b^	95 ± 2^b^
PFT (50 nM) + VK3 (10 μM) + VC (10 mM)	6 ± 2^c^	92 ± 3^c^
SP600125 (1 μM)	2 ± 1	97 ± 2
SP600125 (1 μM) + VK3 (10 μM)	6 ± 2^a^	90 ± 3^a^
SP600125 (1 μM) + VC (10 mM)	8 ± 2^b^	89 ± 3^b^
SP600125 (1 μM) + VK3 (10 μM) + VC (10 mM)	20 ± 2^c^	80 ± 3^c^
NSCI (10 μM)	< 1 ± 0	> 99 ± 0
NSCI (10 μM) + VK3 (10 μM)	3 ± 1^a^	95 ± 2^a^
NSCI (10 μM) + VC (10 mM)	8 ± 1^b^	95 ± 3^b^
NSCI (10 μM) + VK3 (10 μM) + VC (10 mM)	8 ± 1^c^	95 ± 3^c^

Jurkat cells (1 × 10^6 ^cell/mL) were left untreated or treated with specific NF-κB, p53, c-Jun and caspase-3 inhibitors such as ammonium pyrrolidinedithiocarbamate (PDTC, 10 nM), pifithrin-α (PFT, 50 nM), SP600125 (1 μM) and NSCI (10 μM) alone or in presence of VK3 (10 μM), VC (10 mM), VC (10 mM): VK3 (10 μM) at 37 °C for 24 h. After this time, nuclear morphologic and mitochondrial membrane potential changes as a result of NF-κB, p53, c-Jun, and caspase-3 activation were evaluated using acridine orange/ethidium bromide (AO/EB) and DiOC_6_(3) staining, as described in *Materials and Methods*. High polarized and low-polarized mitochondria (green fluorescent DiOC_6_(3) high/low positive cells) and positive apoptotic nuclei percentage is expressed as mean of percentage (%) ± S.D. from three independent experiments. A one-way ANOVA analysis was performed, *p *< 0.0001. ^a^Significantly different from VK3 by Bonferroni *post-hoc *analysis for DiOC_6_(3) and AO/EB. ^b^Significantly different from VC by Bonferroni *post-hoc *analysis for DiOC_6_(3) and AO/EB. ^c^Significantly different from VK3-VC by Bonferroni *post-hoc *analysis for DiOC_6_(3) and AO/EB.

## Discussion

In the present study, we provide for the first time *in vitro *evidence supporting a causative role for oxidative stress in VK3-, and VC-induced apoptosis in Jurkat and K562 cells in a domino-like mechanism involving O_2_^.-^/H_2_O_2_, mitochondrial depolarization, transcription factor activation such as NF-κ, p53, and c-Jun converging in caspase-3 activation and apoptotic morphology. Most importantly, it is shown that high concentration of VC (e.g. 10 mM) alone or in combination with VK3 (e.g. 10 μM) in a ratio 1000:1 and 100:1 induced apoptosis in Jurkat and K562 cells, respectively by a comparable mechanism to VK3 (e.g. 10 μM) and VC (e.g. 10 mM) alone. Moreover, by using antioxidant compounds, we demonstrated that O_2_^.-^/H_2_O_2 _production are essential in VK3-and VC-induced cytotoxicity. This notion was further reinforced by the fact that the antioxidant CP55,940 and NAC completely protect leukemia cells against VK3- and VC-superoxide radicals and H_2_O_2 _toxicity. Most importantly, lymphocytes were more resistant to cell death induced either by VC or VK3 treatment alone than leukemia cells. We speculate that differences in cell cycle (i.e., lymphocytes are in G_o _cell cycle and cancer cells are constantly dividing cells), differences in glutathione content and/or differences in gene expression of antioxidant proteins (e.g., catalase, thioredoxin, superoxide dismutase, glutathione peroxidase) may explain resistance and/or vulnerability in lymphocyte and leukemia cells against VK3 and VC exposure. Taken together our results suggest that different compounds with varied chemical properties might present a similar oxidative stress mechanism of action to erode leukemia cells.

We confirm that VK3 [25] and VC at high concentrations [13,14,26] generate O_2_^.- ^and H_2_O_2_. Moreover, by using antioxidant compounds, it is shown that both reactive oxygen species are related to apoptotic morphology. Our result comply with the notion that H_2_O_2_, as a by-product of O_2_^.- ^dismutation, provokes apoptosis in Jurkat and K562 cells [26]. How H_2_O_2 _might be involved in cell death process? Takada and co-workers [27] have demonstrated that H_2_O_2 _induces NF-κB activation in Jurkat cells through the spleen tyrosine kinase (Syk). Alternatively, H_2_O_2 _may activate NF-κB through phosphorylation IκBα kinase (IKKα) and IKKβ [28]). In accordance with these observations, we found p65-DAB^+ ^nuclei by immunohistochemistry technique in both cell lines treated with either VK3 or VC, as manifestation of p65 activation and translocation to the nuclei, but almost undetectable in untreated cells. Moreover, pharmacological inhibition of NF-κB with PDTC inhibited to almost control value the apoptotic morphology. Taken together these data suggest that VK3 and VC induce activation and translocation of the NF-κB (p65) probably via H_2_O_2_-induced mechanisms. Our results are in agreement with Jones et al. [12]. However, our results differ from Han et al. [29] who showed VC toxicity in HL-60 cells through down-regulation of NF-κB activity. Although the nature of this discrepancy is not known, one possible explanation is that HL-60 cells constitutively express NF-κB (also called "NF-κB positive" cancer cells), which confers cell survival [30]. We conclude that depending on the inducible or constitutive expression NF-κB profile in cells, NF-κB will be an important factor to determine the appropriate therapeutic strategy against lymphoblastic/myelogenous leukemia. Consequently, NF-κB may constitute an important therapeutic target [31]. In accordance with other scientific reports and our present data suggest that activation of NF-κB is linked to cell death signaling in Jurkat and K562 cells [16]. Indeed, NF-κB transcribes pro-apoptotic genes such as p53. Immunohistochemical detection of p53 may indicate that p53 can be directly up-regulated by NF-κB. Moreover, pharmacological inhibition of p53 by PFT completely abolishes VK3 and VC-evoked apoptosis. In contrast to Karczewski et al. [32], our data comply with the notion that p53 is involved in the mechanism of VK3 and VC toxicity in Jurkat and K562 cells [33]. It is noteworthy to mention that p53 transcribes pro-apoptotic genes such as PUMA, Noxa, Bim, Bid, Bax, which are able to permeabilize mitochondria, thus promoting the release of the apoptogenic cytochrome c, which elicits caspase-3 protease activation, leading to nuclear chromatin fragmentation, typical of apoptotic morphology. In fact, caspase-3 NSCI inhibitor protected cells from those noxious stimuli. In contrast to Verrax et al. [15], our data clearly showed that VC, VK3 or VC/VK3 induced toxicity in Jurkat and K562 cells by caspase-3 dependent form of cell death.

According to immunohistochemical staining and pharmacological blockade, we found that VK3 and VC activate c-Jun. How, then, these concurrent signaling pathways are activated either by VK3, VC or H_2_O_2_? As mentioned above, H_2_O_2 _can directly activate NF-κB through Syk or indirectly by activation of the multisubunit IKKα/β by Syk or MEKK1/MAPKK kinase. Therefore, one possibility is that once MEKK1 is activated, it may serve as a cross talk molecule between the JNK and NF-κB pathway [34]. Taken together our findings and this information, it is reasonable to assume that NF-κB and JNK/c-Jun are accomplices with each other during VK3/VC-induced apoptosis in leukemia cells. Furthermore, JNK interacts with p53 in response to stress [35]. We conclude that induction of NF-κB, JNK/c-Jun, and p53 by VK3 and VC might be particularly suitable for the treatment of leukemia [31,36].

During the last decade, VC and VK3 administered in a ratio 100:1 respectively, exhibit synergistic anti-tumor activity by a cell death process denominated autoschizis. This cell death is a type of necrosis characterized by exaggerated membrane damage and progressive loss of cytoplasm through a series of self-excisions [37]. Because cell death is a pure morphological phenomenon [6], we used acridine orange/ethidium bromide (AO/EB) staining as one of the most reliable and unbiased method to identify live, (early and late) apoptotic and necrotic cell [22] compared to other standard methods [38]. Therefore, we first consistently found that (10 μM) VK3 and (10 mM) VC induced (early and late) apoptosis judged against N,N,N′,N′-tetrakis-(2-Pyridylmethyl)ethylenediamine (5 μM, TPEN) reagent, which induces 100% nuclei apoptotic morphology in a caspase-3 and p53-dependent fashion in Jurkat cell line (control, data not shown). We found a ratio 100:1 VC: VK3 induced no cell death and mitochondrial damage in Jurkat cells. However, a significant percentage of (late) apoptotic morphology and depolarized mitochondria in cells were provoked by a ratio 1000:1 (VC: VK3) compared to cells treated with VC and VK3 alone. In agreement with others data, it is found that a ratio 100:1 VC: VK3 induced cell death and mitochondrial damage in K562. Strikingly, Jurkat and K562 cells showed classical apoptosis and necrosis [6] rather than autoschizis morphology [37]. Furthermore, it was found that (1000:1; 100:1) VC/VK3 activates NF-κB, p53, c-Jun and caspase-3, typical of apoptosis. In agreement with Sakagami et al [39] and Ogawa et al. [40], altogether our data suggest that VK3 and VC induce apoptosis in leukemia cells by oxidative stress [41]. In contrast to other reports [15,42-44], our data suggest that autoschizis might not be operative, at least under the present experimental conditions, in Jurkat and K562 cells. Taken together these results imply that VC- and VK3-induced autoschizis is a cell-specific rather than universal cell death process. Altogether our data suggest that a (1000:1; 100:1) VC: VK3 dose should be an intravenously therapeutic dose in the treatment of lymphoblastic and myelogenous leukemia.

## Conclusion

We provide evidence that VK3 and VC alone or in combination induces apoptosis in leukemia cells by a sequential cascade of molecular events involving the production of ROS, simultaneous activation of NF-κB/p53/c-Jun transcription factors, mitochondrial depolarization and caspase-3 activation pathway. These data confirm our hypothesis that VK3 and VC kill leukemia cells by oxidative stress mechanism. Most importantly, VK3 and VC are harmless to lymphocytes, at least under the present *in vitro *conditions. Taken together our results support the notion that oxidative stress may play an important role in the killing of leukemia cells.

## Methods

### Materials

Reagents were purchased from Sigma-Aldrich (St. Louis, MO, USA) if not otherwise specified and were of analytical grade or better. 3,3'-dihexyloxacarbocyanine iodide (DiOc_6_(3), cat # D-273) was obtained from Invitrogen Molecular Probes (Eugene, OR, USA). Ammonium pyrrolidinedithiocarbamate (PDTC, cat. # 548000) and 1,9-pyrazoloanthrone (SP600125, cat # 420119) were acquired from Calbiochem (San Diego, CA, USA).

#### Isolation of lymphocytes

Peripheral blood lymphocytes were obtained from healthy adult (30-40 years old) males' venous blood by gradient centrifugation (lymphocyte separation medium, density: 1.007 G/M; Bio-Whittaker Inc., Walkersville, MD, USA) and cultured as described elsewhere [45].

#### Cancer cell culture

Jurkat clone E6-1 (ATCC^® ^Catalog No. TIB-152™) and K562 (ATCC^® ^Catalog No. CCL-243™) were cultured according to supplier's indications. Cells at 1 × 10^6 ^cells/mL (passage 5-10) were exposed to Vitamin K3 (VK3) and/or Vitamin C (VC) and other products of interest.

#### Experiments with leukemia cell line and lymphocyte cells

Morphological assessment of cell death by fluorescence microscopy using acridine orange/ethidium bromide (AO/EB) double staining

The cell suspension (1 mL, final volume) was exposed to increasing vitamin K3 (1, 5, 10, 20, 35, 50 μM), vitamin C (ascorbic acid at pH 7; 0.1, 0.5, 1, 5, 10, 20 mM) or 10 mM VC: 10 μM VK3 concentrations freshly prepared in RPMI-1640 medium in the absence or presence of different products of interest for 24 h at 37 °C. The cells were then used for fluorescent microscopy analysis as detailed elsewhere [45].

#### Evaluation of intracellular reactive oxygen species

Assessment of superoxide anion radical generation

Superoxide anion radicals were evaluated as described elsewhere [46]. Formazan was quantified under a light microscope (Zeiss Axiostart 50).

#### Assessment of Hydrogen peroxide

H_2_O_2 _was evaluated as described elsewhere [45], except that 2',7'-dichlorofluorescein diacetate (DCFH_2_-DA) was used instead of dihydrorhodamine 123 (DHR-123). Green fluorescent cells (reflecting H_2_O_2 _production) were quantified under a fluorescence microscope.

#### Qualitative analysis of mitochondrial membrane potential (Δψ_m_)

The cancer cell lines were treated as described above and incubated with cationic lipophilic DiOC_6_(3) (1 μM, final concentration) to evaluate Δψ_m _as detailed elsewhere [45]. Green fluorescent cells (reflecting high-polarized and low-polarized mitochondria) were quantified under a fluorescence microscope.

#### Immunocytochemistry detection of NF-κB, p53 and c-Jun transcription factor and caspase-3 protein

The Santa Cruz Biotechnology supplier protocol (goat ABC staining System: cat # sc-2023) was followed for the immunocytochemistry using primary goat polyclonal antibodies NF-κB p65 (C-20)-G (cat#sc-372-G), p53 (FL-393) (cat #sc-6243-G), p-(Ser 73)-c-Jun (cat #sc-7981) and caspase-3 (cat #sc-22171). Immunocytochemistry procedure was performed as detailed elsewhere [45]. DAB^+ ^cells were quantified under a light microscope.

### Photomicrography

The light microscopy and fluorescent photomicrographs shown in figures and supplemental ones were taken using a Zeiss (Axiostart 50) microscope equipped with a Canon PowerShot G5 digital camera.

### Statistical Analysis

The aforementioned parameters were quantified by counting a minimum of 500 total cells blind to experimental setting and viewer. The experiments were performed in 3 independent settings. Data are means ± S.D. of three independent experiments. One-way ANOVA analyses with Bonferroni or Games-Howell post-hoc comparison were calculated with SPSS 18 software. A *p*-value of < 0.05 and < 0.001 was considered significant.

## Abbreviations

AO: Acridine orange; ammonium pyrrolidinedithiocarbamate: PDTC; anion superoxide radical: O_2_^.-^; JNK: c-Jun N-terminal kinase; DAB: diaminobenzidine; 2',7'-dichlorofluorescein diacetate: DCFH_2_-DA; 3,3' dihexyloxacarbocyanine iodide: D_i_Oc_6_(3); dimethylsulfoxide: DMSO; EB: ethidium bromide; hydrogen peroxide: H_2_O_2_; mitochondrial transmembrane potential: Δψ_m_; NBT: nitroblue tetrazolium; 1-(4-Methoxybenzyl)-5-[2-(pyridin-3-yl-oxymethyl)pyrrolidine-1-sulfonyl]-1H-indole-2,3-dione: NSCI; NF-κB: nuclear factor-kappa B; PFT: pifithrin-α; VC: Vitamin C; VK3: Vitamin K3; 1,9-pyrazoloanthrone: SP600125; ROS: reactive oxygen species.

## Competing interests

The authors declare that they have no competing interests.

## Authors' contributions

ARBP performed experiments and analyzed data; MJDR. and CVP. conceived and designed the study, analyzed and interpreted data, wrote the manuscript and all authors gave final approval of submitted manuscript.
